# Maternal DHA Status during Pregnancy Has a Positive Impact on Infant Problem Solving: A Norwegian Prospective Observation Study

**DOI:** 10.3390/nu10050529

**Published:** 2018-04-24

**Authors:** Hanne Cecilie Braarud, Maria Wik Markhus, Siv Skotheim, Kjell Morten Stormark, Livar Frøyland, Ingvild Eide Graff, Marian Kjellevold

**Affiliations:** 1 Regional Centre for Child and Youth Mental Health, Uni Research Health, Uni Research, P.O. Box 7810, 5020 Bergen, Norway; hannec.braarud@bufetat.no (H.C.B.); siv.skotheim@bufetat.no (S.S.); 2 The Office for Children, Youth and Family Affairs, Region West, P.O. Box 2233, 3103 Tønsberg, Norway; 3 Institute of Marine Research, P.O. Box 1870 Nordnes, 5817 Bergen, Norway; maria.wik.markhus@hi.no (M.W.M.); livar.froyland@hi.no (L.F.); ingvild.graff@uni.no (I.E.G.); marian.kjellevold@hi.no (M.K.); 4 The Office for Children, Youth and Family Affairs, Region South, P.O. Box 2233, 3103 Tønsberg, Norway; 5 Department of Health Promotion and Development, University of Bergen, P.O. Box 7800, 5020 Bergen, Norway; 6 Uni Research Health, Uni Research, P.O. Box 7810, 5020 Bergen, Norway

**Keywords:** DHA, pregnancy, infant development, infant problem solving

## Abstract

Docosahexaenoic acid (DHA, 22:6, *n*-3) is a long-chain polyunsaturated fatty acid necessary for normal brain growth and cognitive development. Seafood and dietary supplements are the primary dietary sources of DHA. This study addresses the associations between DHA status in pregnant women and healthy, term-born infant problem-solving skills assessed using the Ages and Stages Questionnaire. The fatty acid status of maternal red blood cells (RBCs) was assessed in the 28th week of gestation and at three months postpartum. The infants’ fatty acid status (RBC) was assessed at three, six, and twelve months, and problem-solving skills were assessed at six and twelve months. Maternal DHA status in pregnancy was found to be positively associated with infants’ problem-solving skills at 12 months. This association remained significant even after controlling for the level of maternal education, a surrogate for socio-economic status. The infants’ DHA status at three months was associated with the infants’ problem solving at 12 months. The results accentuate the importance for pregnant and lactating women to have a satisfactory DHA status from dietary intake of seafood or other sources rich in DHA.

## 1. Introduction

The brain undergoes a rapid growth spurt during the last trimester of pregnancy and the first years of life. Long-chain polyunsaturated fatty acids (LCPUFAs) such as docosahexaenoic acid (DHA, 22:6, *n*-3) and arachidonic acid (AA, 20:4, *n*-6) are important for normal brain functions [1], the development of visual and neural tissues [2,3,4], and subsequently motor and cognitive development [5]. Therefore, adequate levels of DHA and AA are important for pregnant and lactating women, and for infants [6]. The transfer of DHA across the fetal blood–brain barrier depends on the relative amounts of fatty acids in the blood of the fetus, while the transport of DHA to the fetus during this period is highly dependent on maternal dietary intake. Modern diets contain increased *n*-6/*n*-3 ratios (10–25:1), which may influence the effects of polyunsaturated fatty acids (PUFAs) on human metabolism [7]. Thus, while modern diets are AA adequate, obtaining adequate levels of DHA may be a challenge, as large amounts of DHA are needed for the synthesis of membrane phospholipids to complete fetal brain development and neurogenesis [8].

Maternal DHA levels show an overall progressive decline during pregnancy [9]. Thus, unless compensated with higher intakes of seafood or other eicosapentaenoic acid (EPA)- and DHA-rich foods or supplements, stores will be depleted. In addition, we have previously shown that maternal DHA declines after birth and that postpartum levels are determined by DHA status during pregnancy [10]. According to the U.S. Food and Drug Administration’s guidelines, pregnant women are encouraged to consume 8–12 ounces (the equivalent of approximately 226–340 g) of a variety of fish per week [11], and the Norwegian Directorate of Health recommends a daily intake of 200 mg of DHA for pregnant and lactating women [12].

The relationship between seafood and health has been explored in several studies. While earlier research has primarily focused on the health risks associated with contaminants present in seafood, current research also focuses on the potential health benefits from regular fish consumption [13,14]. The nutritional status of EPA and DHA is higher in women consuming oily fish during pregnancy [15,16]. In a large population-based study, high fish intake during pregnancy was associated with improvement in several developmental milestones at eight years of age, even though the association ceased to be significant after adjustment for potential confounding factors [17]. Higher fish intake has also been associated with better cognitive test performance in three-year-olds [18,19]. However, the evidence of the effects of maternal fish consumption during pregnancy on infant cognitive development is not conclusive [20]. Supplementing infant formula with DHA specifically, or in combination with AA, has yielded positive effects on motor and cognitive development in full-term infants [21,22,23], and supplementing breast milk with DHA and AA yielded positive effects on infant problem-solving skills in some studies [24], but not in others [25,26]. Reviews of DHA supplementation trials have also concluded with mixed results. Recent reviews of trials on healthy, full-term infants found that DHA supplementation had limited effects on infant cognition [27], while a meta-analysis of 38 trials, including 53 intervention arms and 5541 participants from pregnancy through infancy, demonstrated an overall significant benefit of *n*-3 PUFA supplementation on infant cognitive development, although not through childhood [28]. 

Maternal and family characteristics, such as socio-economic status, educational level, and stimulation at home, affect both nutritional status and infant development [29,30]. Thus, it is important to control for the effect of socio-economic status when studying the relationship between diet and early child development [31]. While an improvement in socio-economic status may be difficult to accomplish [32], a change in diet during pregnancy and early infancy may be a more realistic target [33].

The majority of the literature draws on intervention studies exploring the association between fatty acid supplementation (i.e., DHA, AA) and cognitive development. However, it is also important to investigate the naturally occurring associations between the fatty acid composition in red blood cells (RBCs) in pregnant women and young infants, and its associations with infant developmental status, in a population-based sample. The brain tissues of interest are generally inaccessible for fatty acid analyses in humans, and the precise DHA intake is difficult to determine. Therefore, surrogate biomarkers are important for defining DHA status [34]. Analysis of RBCs by gas chromatography meets the criteria for a useful, non-invasive biomarker of DHA status. Furthermore, DHA content in RBCs correlates with brain, cardiac, and other tissue levels [35].

Thus, the aim of the present study was to investigate the relationship between the maternal DHA status in RBCs during pregnancy and infant DHA status in RBCs after birth, and its associations with infant problem-solving skills at 6 and 12 months postpartum. 

## 2. Materials and Methods

### 2.1. Design and Study Population

This study drew on data from a prospective longitudinal cohort study on mental health, seafood nutrition, and infant development. The recruitment of participants took place in the Fjell municipality, a (by Norwegian standards) middle-sized municipality in Western Norway from November 2009–June 2011. One-hundred-and-twenty-six mothers gave their informed consent to take part in the study, on behalf of themselves and their infants. The study was conducted in accordance with the Helsinki Declaration of 1975 (revised in 2008). Written informed consent was obtained from all participants, who were free to withdraw from the study at any time, without reason. The Regional Committee for Medical and Health Research Ethics (REC West) Norway and The Norwegian Social Science Data Service approved the study and the consent procedures. A research biobank was established and approved for the storage of biological samples. A description of the background variables of the cohort is given in Table 1.

**Figure 1:**
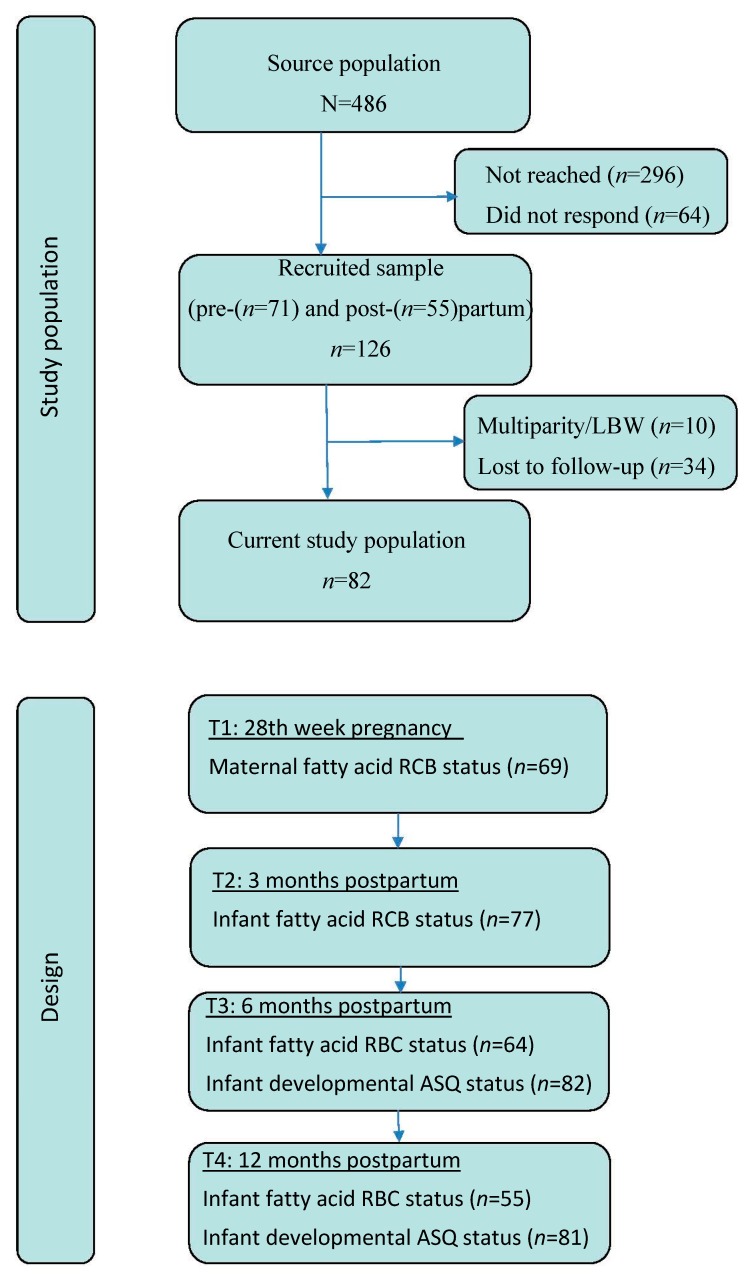
Flowchart depicting study population and design. ASQ: Ages and Stages Questionnaire; LBW: low birth weight; RCB: red blood cell.

The women were recruited by their midwife or medical doctor in the 24th week of gestation (*n* = 71). If not targeted during pregnancy, they were recruited by their public health nurses after birth (*n* = 55). The study involved four waves of data collection: 28th gestational week, three-, six-, and twelve months postpartum (see Figure 1). Ten infants were excluded for low birth weight (birth weight ≤ 2500 g) and/or premature birth (gestational week < 37).

### 2.2. Blood Sampling and Fatty Acid Analyses

Venous blood from the elbow cavity was stored in an ice-water cooled, 4 mL BD Vacutainer^®^ K2E with 7.2 mg vials for preparation of RBCs, which were centrifuged (10 min, 1000× *g*, 20 °C) within 30 min. RBCs were adequately separated to ensure a clean blood fraction. RBC samples were stored at −20 °C for 0–4 weeks prior to transportation on dry ice to a −80 °C freezer, where they were stored until analysis.

The fatty acid composition of total RBCs was determined by ultrafast gas chromatography (UFGC) (Thermo Electron Corporation, Waltham, MA, USA) using a method developed by Araujo et al. [36]. Briefly, 50 µL homogenized samples were mixed with boron trifluoride (BF_3_) and internal standard (19:0 methyl ester), followed by extraction with hexane. The fatty acid composition was calculated using a lab data program (Chromeleon 6.80, Dionex Corporation, Sunnyvale, CA, USA) connected to the UFGC, and identification was ascertained by standard mixtures of methyl esters (Nu-Chek, Waterville, MN, USA). The limit of quantification (LOQ) was 10 µg fatty acid/g samples (wet weight, *w*/*w*). The certified reference materials (CRMs), CRM 162 (soy oil) and CRM 163 (pig fat), controlled the analytical quality of the method and systematic errors. The fatty acid composition of cooking oils was analyzed by gas liquid chromatography (GLC, Trace GC 2000, Thermo Electron Company, Beverly, MA, USA) according to a previously described method [19]. Total lipid content was extracted, filtered, evaporated, and saponified, and fatty acids were esterified. The methyl esters were separated using Auto-GC (Instrument-Teknikk AS, Bergen, Norway), equipped with a 50 m CP sil 88 (Varian, Courtaboeuf, France) fused silica capillary column (i.d.: 0.32 mm), using “cold on-column” injection, a temperature program (60 °C (25 °C/min) to 160 °C (25 °C/min) to 190 °C (25 °C/min) to 220 °C), and flame ionization detector. The fatty acid composition was calculated using a lab data program (Turbochrom Navigator, Version 6.1) connected to the GLC, and identification was ascertained by standard mixtures of methyl esters (Nu-Chek, Elyian, Waterville, MN, USA). Nonadecanoic acid (19:0) methyl ester was used as internal standard. The LOQ was 10 µg fatty acid/g sample (wet weight, *w*/*w*). The results are expressed as absolute and relative amounts. The *n*-3 index was calculated as the content of the EPA and DHA in the RBC membrane, expressed as percentage of total fatty acids [35]. 

### 2.3. Infant Developmental Status

Infant developmental status was assessed with the Ages and Stages Questionnaire (ASQ) (2nd edition) [37]. The ASQ is a parent-completed screening instrument that consists of 19 age-related questionnaires from 4–60 months of age [38]. Each questionnaire consists of five developmental domains (communication; gross motor; fine motor; problem solving; and personal-social) with six questions in each domain. Each domain gives a score from 0 to 60, where a higher score reflects higher developmental skills. Adding together the domain scores gives a total ASQ score from 0 to 300. The parents were asked to describe their child with the possible responses: yes, sometimes, or not yet [39]. The ASQ was translated into Norwegian and validated with Norwegian infants [40,41]. The parent(s) completed the assessment when the infants were both 6 and 12 months old. Because earlier studies have found associations between DHA and problem-solving skills or cognitive development [21,22,23,24], only the problem-solving domain was used in the present study. 

### 2.4. Covariates

We obtained maternal self-reports on age and educational level via a questionnaire designed for the project in the 28th week of gestation and at 3 months postpartum. Length of maternal education was used as a surrogate for socio-economic status in this study, as previous studies have shown that parental education levels are directly associated with child outcomes [42]. Norway is an affluent and egalitarian society when it comes to income distribution. This is why educational level rather than a composite SES measure was used to index socio-economic status (SES) in this study. This approach is also in accordance with the recommendations in the literature [43].

At 3, 6 and 12 months postpartum, the investigator asked the mothers about breastfeeding, in order to determine how long infants had been breastfed. 

### 2.5. Statistical Analyses

Differences between the participants recruited pre- and postpartum were investigated with an independent sample *t*-test, in order to investigate if there were any systematic differences between the two samples with respect to the mothers’ DHA status at 3 months postpartum and the infants’ DHA status at 3, 6, and 12 months postpartum. Normality of the variables was calculated with Shapiro–Wilk statistics. Means (SD) were calculated for the normally distributed variables and median (range) were calculated for the not normally distributed variables. 

Correlations between the mothers’ DHA status in pregnancy and the infants’ DHA status at 3, 6, and 12 months were analyzed using Pearson’s *r*, as these associations were found to be linear. However, as the associations between maternal or infant DHA status and infant developmental score appeared to be non-linear, or monotonic rather than linear, the non-parametric Spearman rank correlation was used to analyze these relationships, using the Spearman rank order concurrently at each age point and as lagged correlations across age levels.

The variables analyzed in this study were both continuous and categorical. Educational level was treated as a categorical variable while maternal age and length of breastfeeding were treated as continuous variables. In order to analyze the relationship between maternal characteristics (i.e., educational level and age), infant nutrition (i.e., months of breastfeeding), DHA status at 3, 6, and 12 months, and infants’ problem-solving scores at 6 and 12 months, Spearman rank order correlations were calculated. Analysis of variance was performed to assess the associations between fatty acid on infant developmental status after controlling for covariates. All statistical analyses were carried out with the use of Statistical Package for the Social Sciences (IMB^®^ SPSS^®^ Statistics 23, IBM Corporation, Armonk, NY, USA).

## 3. Results

### 3.1. Fatty Acid Composition in RBC

Descriptive data of fatty acid composition of RBCs (µg/g RBC and %) in pregnant women in third trimester and infants at 3, 6, and 12 months of age are shown in Table 2. 

### 3.2. Associations between Maternal and Infant Fatty Acids

The mothers’ DHA status (μg/g RBC) in pregnancy was positively correlated with the infants’ DHA status (µg/g RBC) at three (*r* = 0.42, *p* < 0.05), but not at six or twelve months of age. There were also significant positive correlations between the infants’ DHA status (µg/g RBC) at three and six months (*r* = 0.47, *p* < 0.01) of age, while the infants’ 12-month DHA level was not correlated with the infants’ DHA status at either three or six months of age. There were no differences between the groups recruited pre- and post-natally, in either the mothers’ or the infants’ fatty acids status (data not shown).

### 3.3. Associations between DHA Status and Problem Solving Scores

**Figure 2:**
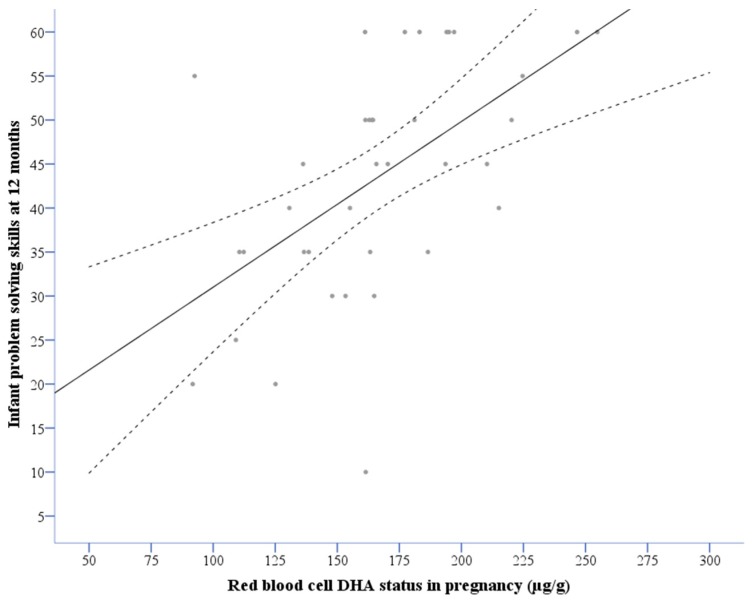
The correlation with 95% confidence interval between maternal red blood cell DHA status in pregnancy and infant problem-solving skills at 12 months. DHA: docosahexaenoic acid.

Infant problem-solving scores at 12 months were significantly correlated with both maternal pre-partum (*r* = 0.58, *n* = 31, *p* < 0.001, see Figure 2) and infant three-month *(r* = 0.26, *n* = 60, *p* < 0.05), but not six- or twelve-month DHA levels. There was a tendency towards a concurrent positive correlation between infant DHA status (µg/g RBC) and problem-solving scores at six months, but not significantly (*r* = 0.25, *n* = 57, *p* = 0.08). There were no other significant correlations between DHA status and other ASQ domains, including the total ASQ score.

There was a significant positive correlation between the mothers’ educational level and the infants’ problem-solving score (*r* = 0.35, *n* = 72, *p* < 0.01) at 12 months, and between the mothers’ age and the infants’ problem solving (*r* = 0.28, *n* = 75, *p* < 0.05) at 6 months. There was also a significant positive correlation between the mothers’ age and the infants’ DHA status at 6 months (*r*= 0.29, *p* < 0.05), and a significant positive correlation between the mothers’ educational level and the infants’ DHA status at 3 months (*r* = 0.31, *p* < 0.05). Thus, the mothers’ education level was treated as a potential confounder in the further analyses. Analysis of variance where the mothers’ educational level was treated as potential confounder showed that mothers’ DHA status (μg/g RBC) in pregnancy was associated with infant problem solving at 12 months (*p* = 0.012). There was also a borderline significant difference in estimated marginal mean problem-solving scores between infants of mothers with the highest educational level (*M* = 49.67) compared to infants of mothers with the second lowest educational level (*p* = 0.051) (Table 3). The model explained 30% (adjusted R square = 0.23) of the variance. 

## 4. Discussion

The main finding in this study is that maternal DHA status in pregnancy was positively correlated with infants’ DHA status at three months and infants’ problem-solving skills at 12 months. Maternal DHA status during pregnancy had the strongest association with infant problem-solving skills at 12 months, and the association was even stronger after controlling for level of maternal education.

The associations between maternal DHA status and infant development found in this study are in line with the results from other studies [21,44,45,46], although Jasani et al. [27] found that most studies on LCPUFA supplementation in healthy, full-term infants had limited impact on infant cognitive development. Thus, our results accentuate the importance of DHA during brain development in pregnancy. 

The present study indicates that the fatty acid composition of the membrane in circulating RBCs during pregnancy is associated with later problem-solving skills. This could be explained by the biological role and rapid accretion of DHA in the human brain during the third trimester and the early postnatal period, a time when the rate of brain growth is maximal [1]. From a developmental perspective, adequate and balanced DHA status in early infancy may benefit the maturation of specific developmental domains that are manifested later in infancy and childhood [44]. Regions of the prefrontal cortex are associated with problem-solving skills [47], and the underlying mechanism for problem solving could represent faster information processing or better attention regulation [4]. In addition, since different parts of the brain have different contents of fatty acids [1] and mature at different time courses [48], we can probably not expect to find a significant association between DHA and problem solving at both 6 and 12 months. The prefrontal cortex involves structures that mature late, and assessment at 12 months may be a more relevant time point to measure problem-solving skills. Therefore, this could explain why the strongest association in this study was between maternal DHA status in pregnancy and infant problem-solving skills at 12 months. In a randomized controlled trial infants who received an LCPUFA-supplemented formula had significantly better problem-solving skills at 10 months than infants who received the no-LCPUFA formula [23].

Parental stimulation and infant home environment were found to interact and determine part of infants’ developmental outcome [44]. The present study found that the mothers’ educational level was positively associated with both the infants’ DHA status at three months and problem-solving skills at 12 months. When we controlled for educational level, the mothers’ DHA status in pregnancy still had a strong and significant association with the infants’ problem solving at 12 months. The results support European Food Safety Authority (EFSA)’s recommended daily intake of 200 mg of DHA for pregnant women. It also fits well with the latest suggested advice from the U.S. Food and Drug Administration [11] and the Norwegian Directorate of Health [12], who recommend seafood intake corresponding to 2–3 dinner meals weekly for all age groups, including pregnant women. Nevertheless, seafood intake among young Norwegian women is low compared to the recommendations [33,49]. In a recent study, using the same cohort of women as in the present study, we found that the median intake of seafood was lower than recommended [10].

The outcomes measured in this study were based on assessments from one instrument only, using parent reports of infant behavior. It could be argued that parents may be biased in judging their own children, which would influence the findings. Thus, the results and their implications should be interpreted with caution. On the other hand, the ASQ has been found to yield accurate developmental information about the child when parents were asked to make judgments about current, observable infant behaviors [37]. There also seems to be a ceiling effect of the ASQ, with less variance among those mothers with highest DHA status in pregnancy, as depicted in Figure 2. Even if the ASQ is a screening instrument, Klamer et al. [50] reported that the ASQ is related to measures of general developmental abilities in preschool children, and that it involves information processing abilities similar to skills measured in older children [51]. Another limitation is the low sample size. The current study population of 82 is only 17% of the source population, with even smaller sample sizes available for the lagged correlations across age levels. This is a limitation, even though we used non-parametric methods to analyze these associations, and that we have previously shown that the DHA status for the mothers and infants that entered the study at three months postpartum did not differ significantly from those that entered the study during pregnancy [33].

## 5. Conclusions

These findings suggest that maternal DHA status in pregnancy is associated with infant problem solving at 12 months. Since higher problem-solving scores in infancy are related to higher childhood IQ scores, this study underlines the importance of adequate maternal DHA status prior to the growth spurt of infant brains in the last trimester of pregnancy. In order to ensure their infants’ as well as their own DHA status, pregnant and lactating women should have a satisfactory dietary intake of seafood or other sources of DHA. Future research needs prospective observational studies with larger samples or randomized-controlled studies to verify the benefits from DHA in pregnant mothers and young infants in relation to infant developmental status. 

## Figures and Tables

**Table 1:** Sample characteristics (*n* = 82).

Characteristics	Value (Min–Max)	*n*
Sex (% boys)	52.4	80
Gestational age (weeks)	39.7 (37–42)	72
Infant birth weight (g)	3611 (2520–4980)	80
Infant head circumference (cm)	35 (32–39)	47
Breastfed at 3 months (%)	67.2	61
Duration of breastfeeding (months)	3.6 (0–6)	61
Maternal age (years)	31 (19–42)	75
Mother’s highest educational level (%)		
Nine-year primary school	4.0	3
High school	30.7	23
1–3 years College/University	34.7	26
4 or more years College/University	30.7	23

**Table 2:** Fatty acid composition of red blood cells (µg/g and %) in pregnant women in the beginning of the third trimester and infants at 3, 6, and 12 months of age.

	Pregnancy		Infants 3 Months		Infants 6 Months		Infants 12 Months	
	(*n* = 32)		(*n* = 60)		(*n* = 53)		(*n* = 47)	
	Mean (SD)		Mean (SD)		Mean (SD)		Mean (SD)	
	µg/g	%	µg/g	%	µg/g	%	µg/g	%
Total SAFA	1103 (107)	39 (2)	1160 (140)	40 (2)	1093 (134)	38 (2)	931 (80)	37 (3)
Total MUFA	526 (105)	18 (2)	565 (156)	19 (3)	504 (82)	18 (2)	492 (90)	19 (2)
Total PUFA	1107 (136)	39 (3)	1082 (115)	38 (2)	1134 (125)	40 (3)	1013 (126)	40 (3)
Total *n*-6 PUFA	816 (123)	29 (2)	825 (102)	29 (2)	801 (118)	28 (3)	739 (114)	29 (3)
Total *n*-3 PUFA	291 (61)	10 (2)	258 (61)	9 (2)	332 (53)	12 (2)	273 (45)	11 (2)
18:2 *n*-6 (LA)	374 (93)	13 (2)	332 (80)	11 (2)	335 (54)	12 (2)	319 (76)	13 (2)
18:3 *n*-3 (ALA)	284 (70)	0.3 (0.1)	6 (3)	0.1 (0.1)	6 (2)	0.2 (0.1)	7 (5)	0.3 (0.2)
20:4 *n*-6 (AA)	319 (49)	11 (2)	372 (51)	13 (2)	348 (54)	12 (1)	290 (48)	12 (2)
20:5 *n*-3 (EPA)	25 (17)	0.9 (0.6)	16 (15)	0.6 (0.5)	18 (16)	0.7 (0.6)	13 (12)	0.6 (0.5)
22:5 *n*-3 (DPA)	53 (13)	1.9 (0.5)	43 (12)	1.5 (0.5)	52 (10)	1.5 (0.4)	38 (9)	1.5 (0.4)
22:6 *n*-3 (DHA)	168 (37)	6 (1)	182 (36)	6 (1)	218 (40)	8 (1)	160 (30)	6 (1)
*n*-3 index % *		6.8 (1.9)		6.9 (1.6)		8.3 (1.7)		7.0 (1.7)

Note: * Sum %EPA + %DHA (% of sum of total fatty acids). SAFA: saturated fatty acids; MUFA: monounsaturated fatty acids; PUFA: polyunsaturated fatty acids; LA: linoleic acid; ALA: alpha-linolenic acid; AA: arachidonic acid; EPA: eicosapentaenoic acid; DPA: docosapentaenoic acid; DHA: docosahexaenoic acid.

**Table 3:** Association between DHA status in pregnancy, mothers’ educational level, and infant problem solving at 12 months.

ASQ Problem Solving 95% Confidence Interval (CI)				
	Coeff.	Lower Bound	Upper Bound	*p*
DHA (µg/g RBC) in pregnancy	5.68 ^a^	1.32	10.08	0.012
Nine-year	−9.94	−19.95	0.07	0.051
High school	−4.46	−14.48	5.36	0.36
1–3 year of college/university	0 ^b^			

Note: ^a^ Increase in ASQ after increasing DHA (µg/g RBC) with 40 (equivalent with standard deviation). ^b^ Set to zero because parameter is redundant. ASQ: Ages and Stages Questionnaire; RBC: red blood cell.
